# Screening with OGTT alone or in combination with the Indian diabetes risk score or genotyping of *TCF7L2* to detect undiagnosed type 2 diabetes in Asian Indians

**Published:** 2011-03

**Authors:** V. Mohan, Jeremy D. Goldhaber-Fiebert, V. Radha, K. Gokulakrishnan

**Affiliations:** *Madras Diabetes Research Foundation & Dr Mohan’s Diabetes Specialities Centre, WHO Collaborating Centre for Non-Communicable Diseases Prevention & Control, International Diabetes Federation (IDF) Centre of Education, Chennai, India*; * *Centers for Health Policy & Primary Outcomes Research, Stanford University, Stanford, California, USA*

**Keywords:** Asian Indians, diabetes, OGTT, risk score, screening, South Asians, *TCF7L2* genotyping

## Abstract

**Background & objectives:**

With increasing number of people with diabetes worldwide, particularly in India, it is necessary to search for low cost screening methods. We compared the effectiveness and costs of screening for undiagnosed type 2 diabetes mellitus (T2DM), using oral glucose tolerance testing (OGTT) alone, or following a positive result from the Indian Diabetes Risk Score (IDRS) or following a positive result from genotyping of the *TCF7L2* polymorphisms in Asian Indians.

**Methods:**

In subjects without known diabetes (n=961) recruited from the Chennai Urban Rural Epidemiology Study (CURES), OGTT, IDRS, and genotyping of rs12255372 (G/T) and rs7903146(C/T) of *TCF7L2* polymorphisms were done. IDRS includes four parameters: age, abdominal obesity, family history of T2DM and physical activity.

**Results:**

OGTT identified 72 subjects with newly diagnosed diabetes (NDD), according to the World Health Organization criteria of fasting plasma glucose ≥ 126 mg/dl or a plasma glucose ≥ 200 mg/dl, 2 h after 75 g oral glucose load. IDRS screening (cut-off ≥ 60) yielded 413 positive subjects, which included 54 (75%) of the 72 NDD subjects identified by OGTT. Genotyping yielded 493 positive subjects which only included 36 (50%) of the 72 NDD subjects showing less discriminatory power. Screening with both SNPs missed 27 (37.5%) NDD subjects identified by IDRS. In contrast, IDRS missed only 9 (12.5%) of the NDD subjects identified by genotyping. Total screening cost for OGTT alone, or with IDRS were 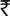 384,400 and 182,810 respectively. Comparing OGTT alone to IDRS followed by OGTT, the incremental cost per additional NDD subject detected by doing OGTT on everyone was 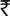 11,199 (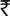 201,590 for detecting additional 18 NDD subjects).

**Interpretation & conclusions:**

For screening a population of subjects without diagnosed diabetes in India, a simple diabetes risk score is more effective and less expensive than genotyping or doing OGTT on the whole population.

India currently leads the world in the number of people with diabetes (51 million) and this number is expected to increase to 87 million by 2030, accounting for one-fifth of the world’s population of diabetes^1^. This rising trend implies a significant health burden due to diabetes in India in the future. Unfortunately more than 50 per cent of individuals with diabetes in India remain undiagnosed. These individuals are at increased risk of developing diabetic complications^2^. Hence there is a need to consider diabetes screening programme alternatives.

Identifying accurate and low-cost screening methods is a necessary first step in assessing the cost-effectiveness of screening to detect undiagnosed diabetes. Evidence on the cost-effectiveness of screening to detect previously undiagnosed diabetes has been considered in other countries including the United States, the United Kingdom, and Australia^3^,^5^. These studies find limited direct evidence for the long-term health benefits associated with early detection of diabetes. However, they also conclude that low-cost screening, targeting high risk groups and improvements in the prevention and management of diabetic complications over the past 10 yr, increase the likely cost-effectiveness of screening. In India, as it is difficult and expensive to screen everyone in the community (universal screening), selective screening of groups with higher prevalence of undiagnosed diabetes may improve the cost-effectiveness of screening. We developed the Indian Diabetes Risk Score (IDRS) using four simple parameters – age, waist circumference, family history of diabetes and physical activity, finding that a score ≥ 60 had optimum sensitivity and specificity for identifying undiagnosed type 2 diabetic subjects^6^.

Although type 2 diabetes mellitus (T2DM) has a strong genetic basis, most candidate genes for T2DM to date have only modest effects, and these associations have been inconsistent^7^,^8^. There is, however, strong interest in developing genetic tests that provide clinical utility^9^. As per Genome Wide Association Studies (GWAS), several new genes have been shown to be associated with T2DM including the transcription factor 7-like 2 (*TCF7L2*) which has been shown to have the strongest association with T2DM to date in several populations^10^–^12^ including Asian Indians^13^. Indeed, the rs12255372 and rs7903146 polymorphisms of the *TCF7L2* gene are now being proposed for routine genotyping of a population to identify T2DM subjects^14^.

The present study was undertaken to compare the effectiveness and costs of screening with oral glucose tolerance testing (OGTT) alone or following positive test results from IDRS, or genotyping of the rs12255372 and rs7903146 polymorphisms of the *TCF7L2* gene, for identifying undiagnosed diabetes in an Asian Indian population-based study.

## Material and Methods

Study subjects were recruited from the Chennai Urban Rural Epidemiology Study (CURES), an ongoing epidemiological study conducted on a representative population (age >20 yr) of Chennai (formerly Madras). The corporation of Chennai city is divided into 10 zones and each zone is subdivided into wards totaling 155 wards and using systematic sampling method, 46 wards were selected. The methodology of the study has been published elsewhere^15^ and is also described in our website: *www.drmohansdiabetes.com*. Briefly, in Phase 1 of the urban component of CURES, 26001 individuals were recruited based on a systematic sampling technique. Fasting capillary blood glucose was determined using a One Touch Basic glucose meter (Life scan, Johnson & Johnson, Milpitas, CA, USA) in all subjects. Subjects were classified as “known diabetic subjects” if they stated that they had diabetes and were on treatment^16^.

In Phase 2 of CURES, all the known diabetic subjects (n = 1529) detected in Phase 1 were invited to the Centre for detailed studies on vascular complications and 1382 responded (response rate, 90.3%). In Phase 3, 1 in 10 of all subjects without known diabetes (n = 2600) were invited to the centre for oral glucose tolerance tests (OGTT) and the response rate was 90 per cent.

For this study, individuals without known diabetes in Phase 3 in whom genotyping of rs12255372(G/T) and rs7903146(C/T) of *TCF7L2* polymorphisms was done using techniques described earlier^13^ were included (n=961). All subjects underwent an oral glucose tolerance test (OGTT). Family history of diabetes, and details on physical activity were obtained using a validated questionnaire^15^. Waist measurements were obtained using standardized techniques^17^. Plasma glucose (PG; glucose oxidase-peroxidase) was measured on a Hitachi-912 autoanalyser (Hitachi, Mannheim, Germany) using kits supplied by Roche Diagnostics (Mannheim, Germany). Diagnosis of diabetes was based on World Health Organization criteria, fasting plasma glucose ≥ 126 mg/dl or plasma glucose ≥ 200 mg/dl 2 h after 75 g oral glucose load^17^ and those detected, were labelled as ‘newly detected diabetic subjects’ (NDD).

The details of the IDRS are published elsewhere^6^ and used the following variables, coded as shown:

1. Age: <35 yr coded 0, 35- 49 yr 20 and ≥ 50 yr 30.
2. Abdominal obesity: males: waist circumference <90= 0, ≥ 90-99 cm 10, ≥100 cm= 20. females: <80= 0, ≥ 80-89 cm= 10, ≥ 90 cm=20.
3. Family history of diabetes: two non diabetic parents 0, one diabetic parent 10 and two diabetic parents 20.
4. Physical activity used three domains and was graded as vigorous 0, moderate 20, and sedentary 30.

A score ≥ 60 provided a sensitivity (72.5%) and specificity (60.1%) for detecting undiagnosed diabetes in our population^6^.

This analysis reports the direct costs of screening and does not include the time and cost of patients travelling to clinical sites and receiving screening. All costs are expressed in 2009 Indian Rupees [INR or 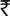]. During this time, the exchange rate per U.S. dollar (USD) was 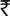 50. Ranges reflect differences in prices of similar services delivered by other organizations in the study area. We assumed that two interviewers would collect data from 50 subjects per day and interviews would be completed in 20 days for 961 subjects. This rate was determined by the time needed per individual screened (5 min to measure weight, height, and waist and 10 min to collect information on family history, gender, and medical background). The salary for two interviewers for 20 days would be approximately INR or 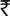 8,000 and the cost of stationery approximately INR or 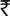 10 per subject, while the cost of IDRS screening for one subject would be 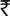 18 (range: 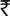 14 to 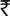 20). Costs for screening with OGTT and genotyping were based on prices charged for services that implicitly include staff time, disposable supplies, and equipment depreciation. OGTT cost 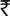 400 per subject (range: 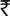 350 to 500). The cost of genotyping for *TCF7L2* SNPs was 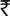 500 (range: 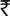 400 to 600) per subject which included the cost of DNA isolation, PCR amplification, genotyping by restriction fragment length polymorphism (RFLP) and additional quality checking of 10 per cent of the samples by different genetic methodology.

### 

#### Statistical analysis:

Fisher’s exact test as appropriate was used to compare proportions. All analyses were done using Windows-based SPSS statistical package (Version 10.0, Chicago).

## Results

Baseline characteristics of study subjects are shown in Table I. Of the 961 subjects screened, 72 were identified with newly detected diabetes (NDD) based on either a fasting plasma glucose of ≥ 126 mg/dl or a 2 h value ≥ 200 mg/dl after a 75 g oral glucose load.

**Table I:** Clinical and biochemical characteristics of study subjects

Variables	Mean ± SD (n=961)

Age (yr)	40 ± 11
Body mass index (kg/m^2^)	23.1 ± 3.9
Waist circumference (cm)	83.1 ± 10.9
Systolic blood pressure (mm Hg)	117 ± 17
Diastolic blood pressure (mm Hg)	74 ± 11
Fasting plasma glucose (mg/dl)	90 ± 26
2hr post plasma glucose (mg/dl)	109 ± 39
HbA1c (%)	5.8 ± 1.0
Serum cholesterol (mg/dl)	179 ± 35
Serum triglycerides (mg/dl)*	106
HDL cholesterol (mg/dl)	43 ± 10
LDL cholesterol (mg/dl)	112 ± 30

Values are mean ± SD; LDL cholesterol, low density lipoprotein; HDL cholesterol, high density lipoprotein;

* Geometric mean

Table II shows the proportion of NDD subjects identified by different IDRS cut-offs as well as the proportions of all study subjects who would require subsequent OGTT. At an IDRS cut off of ≥ 60^6^, the test identified 75 per cent of NDD subjects among the study group (n=54) requiring OGTT screening of 43 per cent of the total population.

Among subjects testing positive for the *TCF7L2* gene polymorphism, 7.3 per cent were NDD (36/493), not statistically distinct from those testing negative (7.7%) (Chi square = 0.01, *P*=0.9, Relative Risk, 1). In comparison, 13.1 per cent of subjects with IDRS score ≥ 60 tested were NDD subjects (54/413), statistically distinct from those testing negative (3.3%) (Chi square = 31.13, *P*<0.001; Relative risk = 4.42).

**Table II:** Screening by IDRS followed by OGTT

IRDS cut-off	Subjects positive on IDRS at each cut-point* (% of 961)		NDD subjects detected via IDRS (% of 72)	
	N	%	N	%

≥10	961	100.0	72	100.0
≥20	951	99.0	71	98.6
≥30	897	93.3	70	97.2
≥40	730	76.0	66	91.7
≥50	604	62.9	62	86.1
≥60	413	43.0	54	75.0
≥70	201	20.9	34	47.2
≥80	58	6.0	12	16.7
≥90	9	0.9	2	2.8

NDD, Newly detected diabetes. An IDRS score of ≥ 60 provided optimal sensitivity and specificity.^6^

* All subjects with IDRS above a given threshold (*e.g*., ≥60) (n=413) would be screened with a subsequent OGTT. Therefore, a number of subjects without diabetes (*e.g*., 359 subjects would be screened with OGTT, and in this sense, were false positives. However, because OGTT was performed for all IDRS-positive individuals, the overall specificity of the combination of tests remained 100 per cent.

**Fig. 1:**
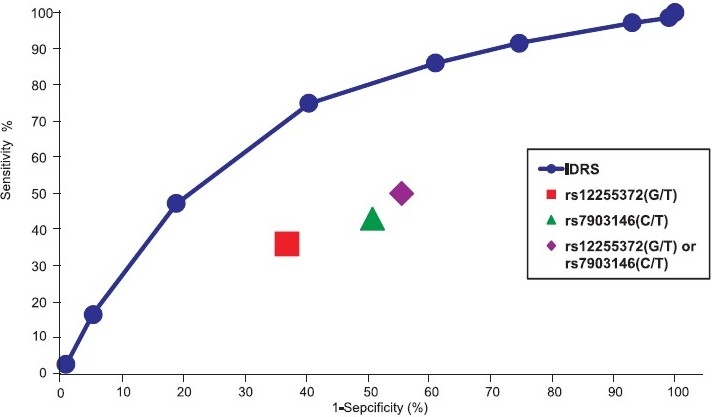
Receiver operating characteristic curve (ROC) showing sensitivity and specificity for detecting previously undiagnosed diabetes using IDRS and two SNPs of *TCF7L2* gene.

Fig. 1 shows a comparison of the sensitivity and specificity of IDRS and genotyping for identifying individuals with undiagnosed diabetes. At a cut-off of ≥ 60, the IDRS has both a higher sensitivity (75 vs 50%) and higher specificity (60 vs 49%) than genotyping (each comparison statistically significant, *P*<0.01). Because all subjects with positive test results on the IDRS or genotyping were also tested with the gold standard OGTT, the specificities of the combined IDRS+OGTT or combined genotyping + OGTT were, by definition, 100 per cent though the lower sensitivities persisted.

**Fig. 2:**
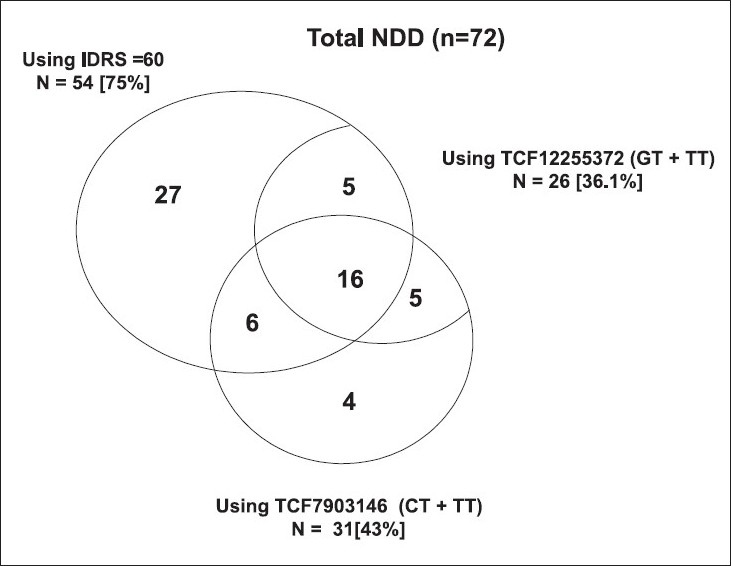
Venn diagram showing the overlap of subjects with newly diagnosed diabetes based on IDRS cut-off ≥60 and two *TCF7L2* SNPs.

Both the IDRS and genotyping miss 9 NDD subjects identified via OGTT Fig. 2. IDRS identified the majority 54 (75%) of NDD subjects that the two *TCF7L2* SNPs identified. The two *TCF7L2* SNPs identified 50 per cent of NDD subjects identified by IDRS. This provides evidence of conditional independence for these two tests.

Given the lack of discriminatory power of genotyping with current SNPs and consequent lack of effectiveness as a screening test we did not assess its costs per incremental case of NDD detected.

The cost of screening all subjects with OGTT was 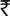 384,400. The cost of IDRS screening for all 961 study subjects was 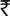 17,610 and required confirmatory testing with OGTT for 413 subjects costing an additional 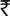 165,200. Comparing OGTT to IDRS screening, OGTT detected 18 additional NDD subjects at an incremental cost of 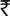 201,590, implying an incremental cost of 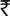
11,199 per incremental NDD subject detected if IDRS is not used.

## Discussion

In this study we report that a simple Indian Diabetes Risk Score (IDRS) is more effective and significantly less expensive for screening for undiagnosed T2DM compared to genotyping TCF7L2 SNPs, the strongest genetic marker for T2DM currently available. Using IDRS screening prior to OGTT, reduces costs while still detecting a substantial portion of NDD individuals.

Genetic susceptibility to T2DM involves a number of variants, each with a modest effect on the incidence of disease in an individual person. The *TCF7L2* gene variants were shown to be strongly associated with T2DM^10^. This was replicated in virtually all populations studied. In monogenic forms of diabetes like maturity onset diabetes of young (MODY) mutation screening could help to identify subjects and this also could be beneficial in terms of therapy (*e.g*., sulphonylureas work better in MODY than biguanides). Studying newer genetic markers may also help in understanding biology and thus in drug discovery. However, currently the effects of most genes for T2DM are modest with odds ratios in the range of 1.1 - 1.5 in most studies^18^. Moreover, compared with clinical risk factors alone, common genetic variants associated with the risk of diabetes had a smaller effect on the ability to predict the future development of type 2 diabetes^19^,^20^. Importantly, genetic testing is currently very expensive for routine population screening. Hence these are more appropriate as research tools than as part of public health programmes or routine screening of the population to identify undiagnosed diabetes.

Asian Indians are highly likely to develop diabetes, and the term “Asian Indian Phenotype” is used to refer to the increased susceptibility of this ethnic group to T2DM^21^. At the same time, many Asian Indians with diabetes are currently undiagnosed. This is one of the first studies to compare the costs of performing a simple diabetes risk score or genotyping prior to OGTT and the use of OGTT for universal adult screening to detect undiagnosed diabetes in a developing country setting. Such studies are relevant because costs can vary considerably between developed and developing countries. For example, manpower costs of conducting IDRS screening would likely be much higher in a developed country.

While this study compares the rates of detection of previously undiagnosed diabetes as well as the direct cost of screening, it does not measure the long-term cost associated with controlling diabetes and preventing complications. It has generally been established that treatment of uncomplicated diabetes is effective in reducing the rates of future diabetic complications. Therefore detecting subjects with diabetes so that they may be properly treated is likely to be cost-effective. Given the potential for differential acceptability of the various screening tests described here and the programmatic challenges of training interviewers to provide IDRS screening, we note the need for additional research assessing the long-term cost-effectiveness of each alternative. We also wish to emphasize that much of the information used to compute the IDRS score is collected as part of standard clinical care. For those individuals who visit their doctor, IDRS may be added to clinical practice with even less cost than what we report. However, as substantial proportions of individuals infrequently visit a physician, outreach-based programmes, particularly in rural areas, will likely be required to achieve early diagnosis for many people with diabetes.

A potential additional benefit of both the IDRS and genotyping is their ability to identify individuals who currently do not have diabetes but are at high risk of developing diabetes in the future. For example, in a previous work, we have shown that the IDRS was the best predictor of incident diabetes. Thus an individual with an IDRS score of ≥ 60 at baseline was three times more likely to develop diabetes in the future than low-risk subjects (IDRS <30)^22^. Future prospective studies are needed to assess the relative risk of developing diabetes among subjects with *TCF7L2* polymorphisms.

We have earlier shown that IDRS helps in identifying subjects with metabolic syndrome and coronary artery disease in the population^23^. In this study, we screened a population of 961 and show that IDRS could identify 75 per cent of newly detected diabetic subjects in this population by screening 42 per cent of the population. Screening of the whole population for diabetes using OGTT is significantly more expensive than IDRS screening. It is possible that the health benefits and averted costs of detecting additional subjects using OGTT are substantial enough to justify this additional cost. However, both the feasibility and affordability of universal OGTT are questionable. Further studies assessing the long-term cost-effectiveness of these various screening methods as well as other methods such as random plasma glucose (RPG) testing are therefore warranted to resolve this question.

Within the medical community there is strong interest in identifying genetic tests that provide substantial clinical utility^9^. With the rapid advances in discovery of T2DM genes, companies have already started marketing DNA-based prediction tests for T2DM^14^. Given that T2DM is a multifactorial disease caused by many different genetic and lifestyle factors, testing of one gene variant is unlikely to provide sufficient information to identify many individuals with undiagnosed T2DM. In addition to identifying prevalent diabetes, studies have shown that several known common risk polymorphisms allow the identification of population subgroups with markedly differing risks of developing type 2 diabetes compared to those obtained using single polymorphisms^24^. The cumulative risk for T2DM depends on other risk factors such as obesity, age, family history of diabetes and lifestyle factors such as physical inactivity. As all these factors are included in IDRS, it is not surprising that IDRS proved to identify more individuals with undiagnosed T2DM than genotyping. Undoubtedly, if the costs of genotyping come down drastically in the future and if more powerful genetic markers are discovered, this scenario could change.

In conclusion, we report that use of a simple diabetes risk score is more effective and less expensive than genotyping and makes it less costly than universal OGTT screening of the whole population to detect subjects without diagnosed T2DM in India.
